# On Crying

**DOI:** 10.1093/oncolo/oyac043

**Published:** 2022-03-17

**Authors:** Tara Sanft

**Affiliations:** Yale School of Medicine, New Haven CT, USA

## Abstract

Is it okay to cry with your patients? This narrative tells a story to help answer this complicated question.

As a breast medical oncologist specializing in cancer survivorship, I have come to appreciate the nuances of patient communication. My multidisciplinary survivorship team have taught me the importance of meeting a patient “where they are at” to maximize the connection and the ability to tailor the information to the individual. I appreciate using communication to create a therapeutic connection so much that I now teach communication skills to the medical students at my institution. In fact, if I reflect back on my career, the privilege of participating in the most intimate conversations of a person’s life is what drove me to pursue dual training in oncology and palliative medicine. In my career as an attending seeing patients in a survivorship clinic, I have realized that most of what I do is listen, normalize, and validate the human experience of diagnosis and treatment of cancer. I truly believe if we can listen and validate our patients’ experiences, we will be healing professionals before we even take out the proverbial prescription pad. Much of the therapeutic relationship has to do with listening for the “emotional story” behind why our patients are in our office. In hearing patients tell their emotional story, we often feel emotions ourselves. It is around this topic that I received a question from a medical student after one teaching session last spring. We were at the tail end of a 3-hour communication class. The first-year medical students had been with me in the fall and now we were finishing the last session in the spring of their first year. They had improved tremendously. They had always been enthusiastic and compassionate, but their ability to use their words to demonstrate curiosity and empathy had grown in them. They were ready for the wards. We had completed all the training and now we were having some casual conversation about communication experiences. “Any questions? You can ask me anything” I had said. “Dr. Sanft, what are your thoughts about crying with your patients?”

Instead of answering, I turned to the group. “Is it ok to cry with your patients?” I asked. They were quiet at first but then one student responded. “It’s important to show emotion with patients, I think, it shows you care.” Someone else added that, while she agreed, it was important that any display of emotion be genuine, not feel forced. Another mused that the amount of emotion might be important, noting you don’t want to “fall apart” in front of patients. This triggered a memory deep inside of me that I hadn’t thought of in years. A time when I felt vulnerable and upset and showed emotion not just because I was devastated about a patient’s outcome, but also because I was full of complicated feelings about the circumstanes.

My turn arrived. *Maybe I’ll share that story,* I thought. I began by stating my opinion: that most doctors felt patients appreciated when their physician showed emotion. It demonstrates a shared experience and can deepen the human connection. In cancer care, we often cannot cure patients and the reality of this is often emotional for both patients and their doctors. Sometimes, holding hands and tearing up is completely appropriate in that moment to share in the disappointment of bad news.

Then I cautioned that there can be limits to how much emotion we show. I told my story. It was nearly 20 years ago, I was an intern in the MICU, completing a 30-hour shift. About 12 hours in, I admitted a 22-year old woman with cerebral palsy and urosepsis. She was hospitalized many times before. She was critically ill, but awake and conversant. I needed to gain venous access, so I attempted a central line in her right neck. I thought I succeeded but I failed to get good return. I told her I needed to try another site. “That’s ok,” she said. “I’m a hard stick. You can try again. You’re doing great.” I was amazed at her encouragement. We talked about her favorite TV shows. She liked sitcoms of all types, but her favorite was *Friends*. She liked the Joey character because he was goofy. We talked about the story lines and our favorite episodes. I searched her TV in the room for a show she could watch while I repeated an attempt for venous access, this time on her left neck. I wanted to use better technique, be more careful, try harder. I didn’t get it. I was embarrassed and frustrated. “It’s ok.” she said. “Try another spot.” As we chatted, I learned that being in the hospital felt pretty routine to my patient. She was used to the staff and the procedures. She admitted it was hard on her parents. She kept a positive attitude and was especially encouraging and kind to me, reciprocating questions throughout the conversation. I eventually obtained access in her groin. It was late at night, and we were behind with our plan for treatment. We started intravenous blood pressure support medications. She was conversational and friendly, bantering with me and the nurse as we explained the medications we were starting and why. Her parents walked in and out of her room, having been through the scenario many times before. Eventually she fell asleep but her vital signs were not stable. Her level of consciousness changed and in the very early morning hours she needed to be intubated.

Dawn broke and she wasn’t stabilizing. I wondered what else we could do. I discussed her case with my resident and fellow. She was very ill and we were doing all we could. I needed to update her parents. I sat with them to describe the situation. And they understood. It was obvious their daughter inherited their gracious attitude. They thanked me for my efforts and decided to stop life support, to allow for a natural death. We were all in the room when she passed away in peace. I remember seeing the sun rise off the horizon as the day team arrived. I was still in her room with her and her parents, progress notes unfinished. I looked at her parents and I felt myself starting to cry. I did more than cry, I sobbed. I sobbed standing in the middle of the MICU, hands over my face, unable to pull it together. Her parents hugged and comforted me. They told me I did everything to help her. I eventually stopped crying. Her parents left and her body was sent to the morgue. I finished rounding. The rest of the day was a blur.

I choked up as I told the students this story. I saw them choke up too. I told them that looking back on it now, as a mother myself, a part of me wishes I hadn’t sobbed to my patient’s parents because it made her loss about me, and what I had just witnessed. But at the same time, I realized I was mourning. I had spent time the night before “chatting” with her while she was stable and this allowed me to care about her more. It made me fight harder to stabilize her, to hope to turn things around. But it didn’t happen and I was devastated.

Thinking about my young intern self, I have a sense of tenderness. It was hard then- I was exhausted. I wasn’t well supported- not because people didn’t care about me but because my co-intern and resident were busy trying to save other peoples’ lives. I felt unsure about my skills, about my ability to turn a critical illness around. I wished my current self could hug my intern self and say*, It’s ok. You’re human. You feel upset because you took the time to know her, understand her likes, her life, her struggles.*

Telling this story to my students showed them how this simple question has an answer that builds on layers of complexity. Yes, it is okay to cry, and if sometimes you sob, then that’s okay too. Being a doctor means facing so many emotions simultaneously: shame, disappointment, sadness, loss, grief, exhaustion. But for me, in this one instance, it also signified the sorrow and the privilege of knowing a patient on the very last day of her life.

